# Iron Supplementation Influence on the Gut Microbiota and Probiotic Intake Effect in Iron Deficiency—A Literature-Based Review

**DOI:** 10.3390/nu12071993

**Published:** 2020-07-04

**Authors:** Ioana Gabriela Rusu, Ramona Suharoschi, Dan Cristian Vodnar, Carmen Rodica Pop, Sonia Ancuța Socaci, Romana Vulturar, Magdalena Istrati, Ioana Moroșan, Anca Corina Fărcaș, Andreea Diana Kerezsi, Carmen Ioana Mureșan, Oana Lelia Pop

**Affiliations:** 1 Department of Food Science, University of Agricultural Science and Veterinary Medicine, 400372 Cluj-Napoca, Romania; i.oanagabrielaa@gmail.com (I.G.R.); ramona.suharoschi@usamvcluj.ro (R.S.); dan.vodnar@usamvcluj.ro (D.C.V.); carmen-rodica.pop@usamvcluj.ro (C.R.P.); sonia.socaci@usamvcluj.ro (S.A.S.); anca.farcas@usamvcluj.ro (A.C.F.); andreeadianakerezsi@gmail.com (A.D.K.); carmen.muresan@usamvcluj.ro (C.I.M.); 2 Department of Molecular Sciences, University of Medicine and Pharmacy Iuliu Hatieganu, 400349 Cluj-Napoca, Romania; romanavulturar@yahoo.co.uk; 3 Cognitive Neuroscience Laboratory, University Babes-Bolyai, 400327 Cluj-Napoca, Romania; 4 Regional Institute of Gastroenterology and Hepatology “Prof. Dr. Octavian Fodor”, 400158 Cluj-Napoca, Romania; magdaistrati@yahoo.com; 5 Faculty of Medicine, University of Medicine and Pharmacy “Iuliu Hatieganu”, 400349 Cluj-Napoca, Romania; ioana.morosan@me.com

**Keywords:** iron, diet, gut microbiota, probiotics, prebiotics

## Abstract

Iron deficiency in the human body is a global issue with an impact on more than two billion individuals worldwide. The most important functions ensured by adequate amounts of iron in the body are related to transport and storage of oxygen, electron transfer, mediation of oxidation-reduction reactions, synthesis of hormones, the replication of DNA, cell cycle restoration and control, fixation of nitrogen, and antioxidant effects. In the case of iron deficiency, even marginal insufficiencies may impair the proper functionality of the human body. On the other hand, an excess in iron concentration has a major impact on the gut microbiota composition. There are several non-genetic causes that lead to iron deficiencies, and thus, several approaches in their treatment. The most common methods are related to food fortifications and supplements. In this review, following a summary of iron metabolism and its health implications, we analyzed the scientific literature for the influence of iron fortification and supplementation on the gut microbiome and the effect of probiotics, prebiotics, and/or synbiotics in iron absorption and availability for the organism.

## 1. Introduction

Iron deficiency is a prevalent problem affecting over two billion individuals around the globe. The main causes of this insufficiency are the lack of iron-rich foods in the diet or the body’s inability to absorb ingested iron owing to acquired or genetic causes. Iron deficiency affects about 40% of the population in developing countries and around 10% of the inhabitants of developed countries [1]. Iron can be obtained through food products, supplements, or medical procedures such as blood transfusion and/or transfusion of red cell concentrates [2]. The most significant concern is not the source of the iron, but the type of iron obtained and its absorption pathways [3,4,5]. Foods such as vegetables, meats, and meat products are proven to be an excellent source of iron, and the fermented version of these products improves dietary iron absorption. Lactic acid, produced by lactobacilli, increases the dietary bioavailability of iron, thus suggesting that utilization of probiotic bacteria can act as a clinical tool for iron bioavailability optimization [6,7,8]. Environmental oxidized iron has low solubility, and for this reason, organisms must develop adequate modalities to compete for the available sources. Human growth and wellbeing are directly proportional to the consumption of this nutrient and are highly affected by the gut microbiota composition [9,10,11]. An increasing number of studies connect iron bioavailability and absorption to microorganism activity in the gut [12,13,14]. Furthermore, the virulence of many pathogens is associated with iron levels [15]. Solely 10% of dietary iron is absorbed in the human body, with the remaining 90% being excreted through fecal matter, thus affecting microbiota balance [16].

In this review, we analyze the scientific literature concerning the influence of iron fortification and supplementation on the gut microbiome and the effect of probiotics, prebiotics, and/or synbiotics in iron absorption and availability for the organism.

## 2. Iron Metabolism: Implications in Health and Disease

**Figure 1:**
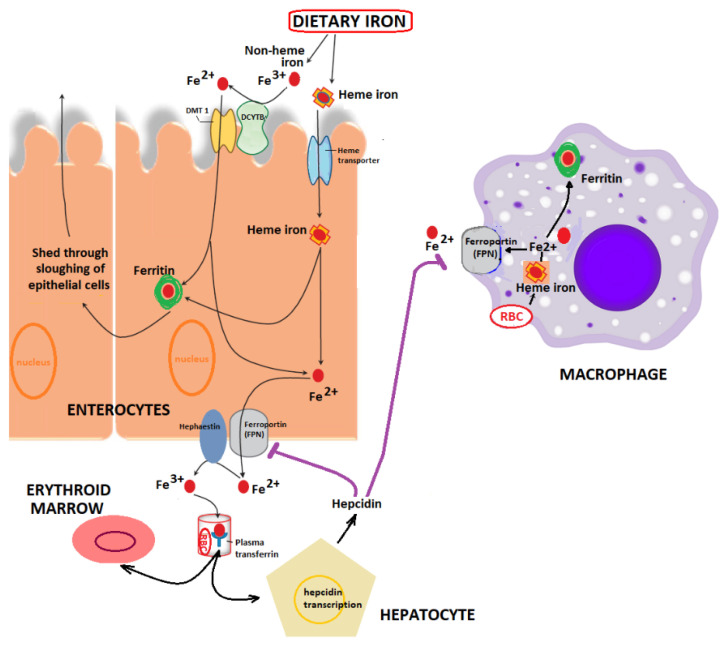
Overview of intestinal iron absorption and metabolism involving enterocytes, hepatocytes, macrophages, and erythroid marrow—adapted from [19,20,21,22,23,24,25,26,27].

Owing to its implication in metabolic processes, iron is essential for all organisms. Its most studied functions are those linked to transport and storage of oxygen, electron transfer, synthesis of hormones, DNA replication, cell cycle control, nitrogen fixation, and antioxidant properties [10,17]. The amount of iron in the body of an adult human male is 3.5–4.0 g, with most of it being found in hemoglobin (Hb) [3]. Iron transport in the digestive system is controlled by several iron-binding proteins (e.g., transferrin, lactoferrin, and bacterioferritin), among other functional agents present at several key sites. Generally, mucins bind to iron owing to the acidic conditions in the stomach, which helps in maintaining it in a soluble state for later uptake in the alkaline conditions of the duodenum. Mucin-bound iron can subsequently pass across the mucosal cell membrane. After entering inside the cells, mobilferrin, the cytoplasmic iron-binding protein, transports it to the basolateral side, where it is exported into the blood plasma. The mechanism of absorption of hem and non-hem iron in the intestinal mucosal cells involves various transport processes and regulatory proteins (Figure 1) [18]. It is at this stage that ferritin levels are considered as the most reliable markers of iron deficiency [17].

Legend: Synthesis of Heme and other metallo-proteins requires >20 mg of iron/day (from diet and mostly by recycling); from this, only 1–2 mg of iron is derived from intestinal absorption (mainly in the duodenum). **Heme** iron from the diet is readily absorbed (through a Heme transporter) and **non-Heme iron** is predominantly found as ferric iron (Fe^3+^). To facilitate the transport of insoluble ferric iron across the luminal membrane of enterocytes, ferric iron (Fe^3+^) is reduced by the enzyme ***ferric reductase duodenal cytochrome B*** (DCYTB) to ferrous iron (Fe^2+^), which is then transported into the enterocyte through the divalent-metal transporter (DMT) 1. Iron’s secretion into the circulation from the basolateral membrane of enterocytes and macrophages uses ***ferroportin*** (FPN). A ferroxidase, ***hephaestin***, is also involved in the export process by converting Fe^2+^ back to Fe^3+^. This step is required for the attachment of iron to ***apo***-***transferrin***, thus forming **transferrin** in plasma. This di-ferric transferrin is the form in which iron is delivered to sites of iron utilization, such as the erythroid marrow, where the red blood cells (RBCs) are produced. FPN is controlled by ***hepcidin*** (from hepatocytes). Binding of hepcidin to FPN causes the latter protein to be internalized and degraded within the enterocyte. Hepcidin, when found in high amounts, inhibits iron release by ***ferroportin***. However, when it is low in concentration, it facilitates iron export into the circulation. In a few cell types (enterocytes, macrophages) iron can be stored as bound to ***ferritin***. In the circulation, iron, which is toxic in the unbounded state, is attached to apo-transferrin (as mentioned above), forming ***plasma transferrin***.

**Figure 2:**
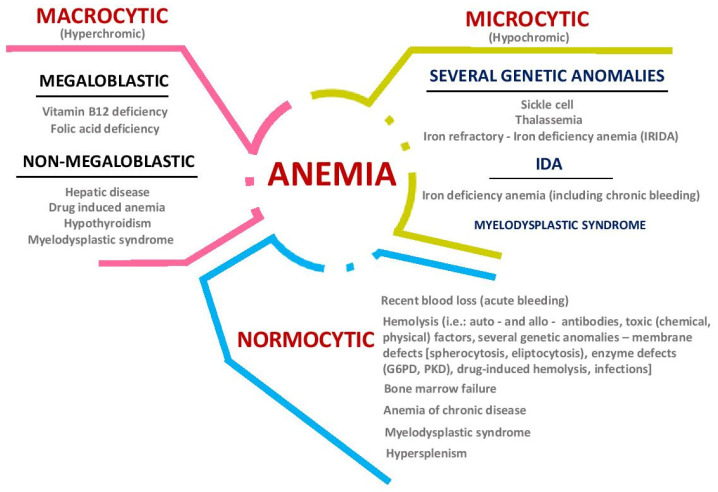
Anemia classification (adapted from [20,32]) (G6PD: Glucose-6-Phosphate-Dehydrogenase; PKD: Pyruvate Kinase Deficiency).

Anemia manifests itself thanks to an inadequate diet or poor iron absorption. An iron deficiency can cause anemia, resulting in the inability to maintain proper tissue oxygenation and body homeostasis [28,29]. About 52% of pregnant women, mainly in developing countries, may develop this deficit. Iron requirements are increased during pregnancy, because of increased necessities; for example, a normal enlargement of red blood cell (rich in hemoglobin) diameter can be observed [3,30]. Regarding the prevalence of iron deficiency anemia (IDA), the World Health Organization (WHO) reports that about 50% of all anemia cases are caused by iron deficiency [31]. A classification of anemia (based on red blood cell morphology) is presented in Figure 2.

.

Other classifications are based on criteria such as erythrocytes’ production, nutrition deficiencies (iron, proteins, folate, vitamin B12, and vitamin B9), genetic deficiencies in synthesis of Hb chains, red blood cells cytoskeleton with abnormal shapes, or based on secondary pathologies including chronic bleeding [18]. In some cases, sedentary lifestyles, diabetes, and obesity are also linked to anemia [18,33,34].

According to WHO [31], anemia caused by iron deficiency can be classified according to three stages, ranging from the first (mild form) to the third stage (severe anemia) with low Hb (<70 g/L for children under 5 years and pregnant women, and <80 g/L in children over 5 years, adolescents, and adults). This last type will affect the cognitive and physical functions of the individual, resulting in the patients presenting chronic fatigue and lower work capacity [9,35]. However, it can be corrected through diet or with iron-based treatment. The most common causes of iron deficiency are low solubility of iron, low bioavailability, and loss of blood due to hemorrhage [6]. The explanatory factor for these conditions is that most of the iron from ingested food has low solubility and, consequently, low bioavailability [1]. Once absorbed, iron enters into the systemic circulation and becomes available either for storage or for the physiological functioning of the body [36].

## 3. Gut Microbiota and Iron Deficiency

The human gastrointestinal microbiota has an essential role in metabolism and cognition, thus influencing vital functions of the human body. The balance in our diet and nutrients influences the healthy growth of the microbiota in the first years of life [37,38,39,40]. In order to modulate the gut microbiota balance and avoid a dysbiosis, probiotics and prebiotics can be administered orally, by fecal microbiota transplant or by other methods [37]. Probiotics are live microorganisms that, once introduced in the gut, improve its microbial composition. Prebiotics are selective non-fermented food ingredients that bring changes in the microbial activity by feeding the probiotics, and thus providing many benefits to the host [41].

The intestinal microbiota of adults and children differs depending on several factors such as age, ethnicity, geography, and lifestyle [38]. A healthy diet that contains whole grains, fresh fruits and vegetables, nuts, and medicinal foods helps us fight various diseases and maintain a proper balance of the gut microbes [11,42]. Although physiological and neurological consequences of iron deficiencies have been addressed extensively, further studies need to evaluate their impact on development of gut microbiota [43,44]. The absorption of iron, which can result either from diet or oral supplements, is a complex mechanism containing many reactions. The literature surrounding mucosal iron absorption [45,46,47,48] is controversial in the debate of iron absorption, presenting unclear explanations of whether it is absorbed in the ferrous or ferric form. However, it was agreed that the key role of iron absorption is the intestinal solubility of the oral medication [49]. According to a study, during the weaning process of different babies from Africa, the gut flora was changed by the fortification of iron, having as consequences a reduction in the number of *Bifidobacteria* as well as an increase in the *Enterobacteriaceae* and some specific enteropathogens (e.g., *E. coli* pathogenic). Moreover, in the same study, it was mentioned that the fortification of iron increased the faecal calprotectin levels, showing the inflammation of the intestine [34]. It is established that, when the iron levels drop, intestinal infections can occur because of the influence on the gut microbiota composition [50]. A study conducted on young women in south India correlated a low iron level with low levels of lactobacilli in the feces [51].

Both deficiency and excess of iron levels are important in terms of gut microbiota dysbiosis and the appearance and development of inflammation and colorectal cancer [42,52,53]. There are specific bacteria that mediate the development of intestinal pathologies such as *Streptococcus bovis*, *Enterococcus faecalis,* and *Clostridia*. They release hydrogen sulfide and secondary biliary salts, which accelerate inflammation and carcinogenesis [54,55]. Some bacteria, including *Bifidobacterium longum* and *Lactobacillus acidophilus,* prevent intestinal inflammation [56]. According to [57], gut inflammation was not influenced by the high amount of additional iron (50 mg Fe, 4 days/week) for 266 days and the concentration of the main microorganisms and short chain fatty acids (produced by the probiotic species) in the fecal matter was unchanged. A previous study, conducted on rats by the same authors, showed that iron-deficient rats had considerable lower concentrations of butyrate and propionate (produced specially by *Roseburia* spp./*E. rectale* group) in the cecum and the gut microbiota composition was strongly modified [58]. This implies that iron deficiencies have a notable impact on the gut microbiota, while iron excess does not.

It was determined that the alteration in a host’s iron homeostasis may affect “the luminal iron content of the intestine, and thus the composition of intestinal bacteria” [59]. An analysis of mice feces demonstrates that the iron regulatory protein 2 (Irp2) and the protein of the mutated genes that appears in diseases such as hereditary hemochromatosis Hfe (affected in hereditary hemochromatosis) are highly involved in iron regulation. The test results demonstrated that, compared with the wild type mice control, the microbiota composition has significantly changed in *mutant* mice (Irp2-/- or Hfe-/-). Therefore, the iron metabolism in the host highly affects the type of host gut microbiota [59]. Moreover, the pH of the colon contents is a factor that dictates iron absorption. For example, microorganisms can ferment galacto-oligosaccharides, leading to a decrease of the pH in the intestine, and thus facilitating iron absorption at this level. Therefore, the acetic acid containing products made by probiotics can be introduced into the diet, and could improve iron absorption [60].

According to a study, fluctuations of iron concentrations can have pathological effects, negatively influencing the intestinal microbiota composition. Compared with germ-free mice colonized with gut bacteria, in germ-free mice, an 8- to 10-fold increase in metal transporter protein 1 (DMT 1) and DCYTB expression and a twofold reduction in ferroportin expression in the duodenum can be observed. The enterocytes of germ-free mice were low in iron and, after colonization with gut bacteria, the epithelial cells favorized iron storage [61]. The mechanism is explained in a study conducted by Gonzales et al. 2017 [62]. They demonstrated that *Lactobacillus fermentum*, a main probiotic found in human microbiota, exhibits a remarkable ferric-reducing activity. P-hydroxyphenyllactic acid, a metabolite produced by this strain, increases iron absorption through the DMT 1 transporters from enterocytes by reducing the Fe^3+^ to Fe^2+^. Similar results can be found in a study (on Kenyan infants) that showed how iron-fortified powder can negatively influence the gut microbiota composition by increasing the number of the pathogens, thus causing intestinal inflammation [34].

In terms of genetics, different types of hereditary hemochromatosis are caused by mutations in the HFE, hepcidin (HAMP), hemojuvelin (HJV), ferroportin (SLC40)A1, and transferrin receptor 2 (TfR2) genes that correspond to genetic disorders of iron overload [63,64]. Ferroportin is an exporter of hepcidin, the hormone that regulates iron in the body, which is affected by mutations in the HAMP gene [65,66]. Intestinal epithelial and fecal metal contents are directly influenced when a lack of proper function of ferroportin occurs because this protein is involved in iron export into circulation from both enterocytes and macrophages [67]. Mutations in iron regulatory gene SLC40A1, which encodes ferroportin, cause a hepatic iron overload hemochromatosis phenotype that negatively affects the intestinal microbiota [68,69].

An iron-rich environment in the gut is conducive to *Proteobacteria*. Studies in children have shown that excess iron causes inflammation and the growth of pathogenic bacteria [34,70].

Inflammatory bowel disease leads to a lack of iron absorption. Iron excess from the intestinal environment can create pathobionts (potentially pathological cells) that are a result of the transformation of commensal microbiota bacteria [71]. Colorectal cancer may be induced by these pathobionts, which can also induce inflammatory bowel disease [72].

## 4. Iron Absorption from Supplements and from Foods

Although iron is an essential nutrient in our diet, iron deficiency is the most frequent nutritional disorder worldwide. Iron availability, in heme or non-heme form, in various foods such as legumes, milk, meat, and juice, was demonstrated many years ago [73]. Iron absorption in the intestine occurs by different pathways depending on the type of iron present. Absorption of heme iron (Fe^2+^), predominantly found in the meat diet, is partially known and probably mediated by a heme carrier into the enterocytes. In the cells, iron is released from heme by hemoxygenase-1 (HO-1). Non-heme iron (Fe^3+^), predominantly present in the vegetarian diet, is reduced by ferric reductase CYBRD1 (DCYTB) enzyme to Fe^2+^ before being transported into the intestinal absorptive cells. Once non-heme iron is reduced to heme iron in the intestinal lumen, the latter is transported into the intestinal enterocyte by a protein called the divalent metal transporter (DMT 1). Inside the enterocytes, iron can be either stored as ferritin or transported through the basolateral membrane into the bloodstream by ferroportin (FPN1), also called the iron exporter. Hepcidin regulates the FPN1 expression. Hephaestin modulates the metabolism and homeostasis for iron absorption, namely by integrating two Fe^3+^ into one transferrin molecule (Tf) and linking iron with apo-transferrin.

There are many options regarding the treatment of iron insufficiency, including oral iron derivates, which can effectively get absorbed in the intestine and mostly in duodenum, taking into consideration that the stomach and the other parts of the gut are less involved in absorption processes [49].

It has been shown that adding meat and juice to legumes increases the availability of iron because vegetables contain protein and minerals, but are limited in ascorbic acid. Milk had the lowest iron absorption rate owing to a large amount of calcium present. In order to increase the iron absorption, pregnant women and children should consume foods with as much ascorbic acid as possible, but not simultaneously with milk [74]. In a study, mice were tested by giving them water, green tea, black tea, or tea extract while consuming iron-based diets. The results showed that both black and green tea do not pose a risk with respect to the availability of iron and do not adversely affect the growth or metabolism of rats, but the interaction of iron absorption in humans with these tea compounds should be studied more closely [75]. Iron accumulates in the body from the first days of life, and when there are no genetic defects, its absence from the body signifies the presence of unhealthy foods in the first months of life or in the mother’s diet. It has been reported that iron-fortified formulas are beneficial in preventing this deficiency. There are studies on newborns (up to maximum 32 weeks of age) that have followed the two categories of nutrition: those who received iron fortified formulas and those fed with their mother’s milk. Iron retention was calculated every week (Hb). Statistics have seen a balance of iron in the body as hemoglobin from birth and hemoglobin from the first 3–6 months was not considerably changed between infants fed with milk formula and those fed with human milk [17].

There are several microelements and metabolites, such as ascorbic acid (vitamin C), folic acid, citric acid, peptides rich in the amino acid cysteine, and vitamin A, that enhance intestinal iron absorption [76,77,78,79]. Some of these molecules can act indirectly, by abolishment of the inhibitory action of phytates found in coffee and tea [80]. Polyphenols (anthocyanidins, flavones, flavanones, isoflavones, and isoflavanones) inhibit the non-heme iron absorption from food. Alcohol simultaneously inhibits the ferrous iron absorption and increases the absorption of ferric iron [18]. It is also shown that the administration of probiotics and prebiotics may increase iron absorption [6,60,81,82].

Iron deficiency anemia impacts people of all ages worldwide. Thanks to the severity and high prevalence of this problem, governmental organizations implemented strict regulations to ensure proper iron nutritional needs. Fortification of foods with iron has been a common practiced in recent decades. However, the greatest concern is the physiological availability of iron ingested from fortified foods. Foods, acting as iron carriers, must be tailored considering their synergistic influence with iron complexes for absorption and increased availability. However, in this approach, there are several issues that need to be solved in terms of large-scale applications, economic efficiency, safety, and consumer acceptability [18].

Iron fortification is a process that includes the addition of vitamins and minerals, which are essential micronutrients used to improve the food quality from a nutritional perspective. To overcome iron deficiency anemia, affected individuals should consume foods that are rich in micronutrients. Microorganisms developed the siderophores (high-affinity iron-chelating compounds, secreted by microorganisms) system, a mechanism for obtaining iron from the environment. However, the excess of iron affects microorganisms’ pathogenicity; thus, to minimalize the negative effect of iron fortification, a better approach should be created. Moreover, iron is absorbed in the presence of fermentable carbohydrates that stimulate “the growth of bacteria that produces short-chain fatty acid such as propionic acid”, which increases mineral intake [83]. Micronutrient deficiencies are mostly observed in impoverished populations. A possible solution for this problem could be the supplementation of micronutrients to increase the nutritional status. An example of such supplementation is multiple micronutrient food supplements [52], which include vitamins such as B1, B2, B6, B12, and A, as well as calcium, iron, and lysine. “Ferrous sulfate is the most bioavailable form of iron” [59], and its interactions with micronutrients can lead to an increase of absorption.

The main foods used for iron fortification are cereals and dairy products and, to a smaller extent, salt, sugar, and condiments. Using cereals, their flours, and derived food products as iron carriers is disadvantageous because of their high phytic acid content, which can diminish iron absorption [1]. Unabsorbed iron destroys the gut microbiota and causes changes in the ratio of protective and pathogenic bacteria. It is thus recommended to treat this deficiency intravenously [56].

Fortified iron foods using probiotics could help reduce anemia. This technique has several advantages because it is less costly, more accessible, and reduces the use of medicines. By developing this emerging approach for mitigating micronutrient deficiency, precision nutrition can be achieved [74]. Precision nutrition helps group people according to the iron content in their body, thus allowing the recommendation of targeted diets that are rich in iron, leading to a decrease of anemia frequency in the population [13].

For food scientists, the greatest and most important challenge is to provide food for the growing population, while considering the content of macro and micronutrients. Hence, several procedures need to be tailored to properly increase the iron content and reduce inhibitors in food crops. Moreover, it is necessary to study the effect of some food additives, such as prebiotics, probiotics, and metal chelators [36].

## 5. Probiotic, Prebiotic, and Synbiotic Approach in Iron Deficiency Treatment

### 5.1. Probiotics and Iron Deficiency Treatment

Considering that the hazard of iron deficiency for the worldwide population is of high importance, it is crucial to implement appropriate strategies to combat this problem. The most frequent strategies are nutrition programs; iron supplementation in foods; iron drug supplements; and probiotic, prebiotic, and symbiotics approaches [30,33,60,73].

A systematic review demonstrated how the use of *Lactobacillus plantarum 299v* helps in the prevention of iron deficiency anemia. It has been found that this probiotic improves dietary non-heme iron absorption in active Caucasian Europeans [6]. Rosen et al. [81] also uses *L. plantarum* 299v for the treatment of iron deficiency in pediatric patients, but with no favorable results for iron absorption. Low-dose ferrous sulfate (1–3 mg/kg/day) was administrated to children, with or without the probiotic. The researchers observed no significant difference regarding serum ferritin level in subjects taking the probiotic *L. plantarum* 299v when compared with controls. No association was made regarding ferritin level and probiotic use.

Another study, performed on rats, investigated the effects of oral multispecies probiotic (*Bifidobacterium bifidum* W23, *B. lactis* W51, *B. lactis* W52, *Lactobacillus acidophilus* W37, *L. brevis* W63, *L. casei* W56, *L. salivarius* W24, *Lactococcus lactis* W19, and *Lc. lactis* W58 in equal proportions), administrated in low 2.5 × 10^9^ and high 1 × 10^10^ dosages. The result revealed that a single log increase in the dosage influences the total counts of probiotics in the feces. Concerning the iron-binding capacity, it was higher in the high dose probiotic group when compared with the control group. Both high and low dose probiotic groups manifested low levels of serum iron, thus high iron levels in the liver, pancreas, and duodenum were registered in the high dosage probiotic groups [82].

Body inflammation alters nutrient’s availability, including iron, as a mechanism for microbial growth limitation. One of the leading causes of acute gastroenteritis is *Salmonella enterica serovar typhimurium* (*S. typhimurium*). Recent studies have shown that *S. typhimurium* flourishes in the inflamed gut while hunting for iron siderophores. By administering a probiotic strain, *Escherichia coli* strain Nissle 1917, that uses similar mechanisms to assimilate iron, *S. typhimurium* colonization in mouse models was reduced. *E. coli* Nissle probiotic activity hangs on the iron acquisition, given that mutants lacking in iron uptake colonize the intestine, but do not reduce *S. typhimurium* counts in the feces. Iron availability influences *S. typhimurium* colonization and *E. coli* Nissle reduces *S. typhimurium* intestinal growth by competing for this restricting nutrient [84].

Table 1 shows most relevant studies related to iron absorption in relation to single or multiple probiotic strains administration.

### 5.2. Prebiotics and Synbiotics in Iron Deficiency Treatment

Prebiotics are functional food components that stimulate the growth and colonization of beneficial bacteria in the gut, and ultimately improve the health of the body. A noteworthy role in intestinal physiology is played by gut microbiota colonization. To effectively reduce the risk of certain diseases (cancer, lipidemia, and so on), we can take advantage of prebiotics such as galacto-oligosaccharides, fructo-oligosaccharide, inulin, and pectin. These components help to implement proper gut microbiota colonization and to maintain its balance [41]. Pre- and probiotic consumption is called symbiosis.

Probiotic fructans (selective carbon sources) fortify the health of the hosts. They influence blood parameters, such as decreased blood urea, uric acid, ammonia, and nitrogen balance. Several studies that have been conducted claim that fructans such as inulin have beneficial effects on the body’s colon functions, even improving minerals’ absorption. There are two types of physiological effects: direct effects within the large intestine and on the intestinal microbiota and indirect systemic effects that influence the metabolism and reduce the risks of disease [94].

Several studies correlated prebiotic and/or synbiotic intake to an increase of iron availability, mostly by converting Fe^3+^ to Fe^2+^ owing to their ferric-reducing activity, and promoting iron uptake by enterocytes. Most of these studies are summarized in Table 2.

In one study, pectin was assembled to iron nanoparticles as matrices for delivery of *L. plantarum* CIDCA 83114. Scientists investigated the biophysical stability of iron–pectin nanoparticles and analyzed their effect. The results showed that iron is not toxic for the probiotic cells, and did not affect the bacteria’s viability, which means that it is efficient in both iron delivery and bacterial stabilization. This can also be used as an alternative solution to overcome iron deficiency [101]. An experiment on Sprague–Dawley rats showed that the intake of non-purified prebiotic galacto-oligosaccharides for 3–4 weeks might enhance Ca, Mg, and Fe absorption [108].

Another study aimed to synthesize organic compound derivatives from polysaccharides ready to bind iron to help in iron deficiency treatment. An inulin-succinic anhydride–cysteine–ferric chloride complex was obtained, having good biodegradability in the presence of inulinase and high muco adhesion properties. The results revealed that the potential employment of such complexes in the oral treatment of iron deficiency anemia or as a supplement of iron-fortified foods can be utilized [109].

A study revealed that synbiotic treatment could improve the body’s iron absorption. The study was conducted on primary school children (9–12 years) for 3 months. They were divided into two groups: those who consumed iron supplement as syrup (given twice a week), and those who consumed a synbiotic mixture—*Lactobacillus plantarum* Dad 13 and fructo-oligosaccharide in fermented milk (six times per week). As a result, the children did not present significant differences because they came from wealthy families and did not suffer from food shortage. However, a greater presence of *E. coli* bacteria was observed among those who drank only iron supplement syrup, and a high number of Bifidobacteria was counted in the stool of those who consumed the synbiotic formula with fermented milk [33].

Another article examined the in vitro effects of inulin on the availability of iron in two probiotic yogurts (milk- and soy-based yogurts). The evaluation was made before and after yogurts’ incubation, monitoring the dialyzable iron fraction, the cell ferritin formation, and cell associated iron. The Caco-2 cell line is a line of human adenocarcinoma cells that is a useful model for studying iron absorption, used in the mentioned study. After incubating both yogurts with and without inulin, the formation of ferritin was higher. This demonstrates that inulin by itself does not directly influence iron absorption, but has a positive impact when used by the probiotic cells [110].

Nanoparticles, such as iron oxide nanoparticles, are a further alternative to dietary supplements. They simultaneously retain the organoleptic properties in food. An innovative formulation is composed of iron oxide nanoparticles, pectin, and lactic acid bacteria. These three components assembled could offer the best form of protection for the microorganisms and for the administration as well as insurance of the safe arrival of soluble iron to the intestine [101].

A different study examines the effects of synbiotic intake on patients with type 2 diabetes as many metabolic complications are associated with changes in serum minerals. The intake of synbiotics can have positive effects on nondiabetic patients and on type 2 diabetic patients, showing significant changes regarding serum iron absorption [111]. However, extensive studies need to confirm the probiotic, prebiotic, and synbiotic positive effect in iron deficiency treatment. Specific recommendations regarding strain, dosage, synbiotic iron formulations, and metabolic implications need to be clarified.

## 6. Conclusions

Iron metabolism involves many processes and, even excluding the genetic defects, there are several causes for iron deficiency in humans. Therefore, multiple approaches for increasing iron absorption exist. Probiotics, prebiotics, and synbiotic formulations can be employed to achieve the desired iron concentration. Nowadays, studies reveal multiple mechanisms through which these formulations can help in regulating the iron deficiencies. Understanding probiotics’ capacity to act as carriers for iron, to convert unavailable iron into its available form, or to create metabolites that indirectly increase the iron absorption in the gut is essential in this approach. These formulations need to become the focus of further studies because current studies, although shortcoming in number, already have promising results. Gut microbiota modulation through pro- and prebiotic intake can affect the iron absorption, but the type of ingested iron is also essential. Cytotoxicity of the unabsorbed iron on the gut cells (enterocytes) needs to be extensively addressed and tailored foods can bring an outstanding approach in iron deficiency treatment.

## Figures and Tables

**Table 1:** Probiotic intake in different types of iron deficiencies and their effect on the iron level in the organism. IDA, iron deficiency anemia.

Type of Iron Deficiency	Probiotic Strain	Type of Administration	Results	References
Low iron absorption	*Lactobacillus. plantarum* (FS2)	Orally; non-alcoholic sorghum-based beverages	↑ iron bioavailability by 128–372% in the fermented beverages	[85]
Anemia (IDA)	*L. plantarum* 299v	Orally; pearl millet seeds	↑ iron absorption	[86]
Anemia (IDA)	*Streptococcus thermophilus*	Orally; fermented milk beverage	↑ iron absorption and utilization (amelioration of blood hemoglobin, serum iron, total iron binding capacity, ferritin)	[87]
Anemia (IDA)	*L. fermentum*	Orally; nanoparticles	probiotic internalize into the enterocyte delivering the nanoparticles and providing an adequate iron level	[88]
Anemia (IDA)	*L. fermentum*	In vitro	↑ iron absorption	[62]
In menstruation	*L. plantarum* 299v	Orally; capsules, with a meal with a high iron bioavailability	↑ iron absorption when administered together	[89]
Anemia (IDA)	*L. plantarum* 299v	Orally; fruit drink	↑iron absorption	[7]
Anemia (IDA)	*L. acidophilus*	Orally; fermented bread	↑ ferritin formation significantly in the intestinal cells (in vitro) and animal serum (in vivo) ↑ iron absorption	[90]
Anemia (IDA)	*L. plantarum* 299v	Orally; capsules together with iron and vitamin C	↑ iron level in the blood	[91]
Low iron bioavailability	*Bifidobacterium bifidum* and *B. longum*	Orally; powder follow-up infant formulas	↑ apparent iron absorption or retention (*p* < 0.05)	[60]
Iron deficiency	*L. plantarum* 299v	Orally; capsules	The treatments were well-tolerated, with mild side effects No significant difference in the increase in serum ferritin in children	[81]
Healthy	*L. plantarum*	Orally; mix of raw vegetables	↑ bioavailability of iron	[92]
Abnormalities of iron metabolism related to obesity	Probiotic mixture (*B. bifidum* W23, *B. lactis* W51, *B. lactis* W52, *L. acidophilus* W37, *L. brevis* W63, *L. casei* W56, *L. salivarius* W24, *Lactococcus lactis* W19, and *Lc. lactis* W58)	Orally; powder	Multistrain probiotic supplementation may influence iron metabolism in obese postmenopausal female patients; further studies are needed	[93]
Anemia (IDA)	*Lactobacillus plantarum* Dad 13	Orally; fermented milk	No difference on the iron status, height, weight, and gut microbiota profile	[33]

“↑”- increase.

**Table 2:** Prebiotic and synbiotic intake in different types of iron deficiencies and their effect on the iron level in the organism.

Type of Iron Deficiency	Prebiotic/Synbiotics	Type of Administration	Results	References
Anemia (IDA)	Galacto oligosaccharides and inulin	Orally; wheat flour	Improved immune function of iron-deficient women	[95]
Healthy	Inulin	Orally; supplement	Improved the iron and anthropometric status	[96]
Iron deficiency (ID) and IDA	Fructo- and galacto oligosaccharides	Milk-derived products	Improved iron bioavailability	[97]
Anemia (IDA)	Inulin	Bioyoghurt—inulin and iron salts	↑ ferric sulphate bioavailability ↓ calcium bioavailability inulin addition significant ↓ iron bioavailability	[98]
Anemia (IDA)	Bifidobacterium lactis HN019 and oligosaccharides	Orally; milk	↓ risk of anemia and iron deficiency and helped to gain weight	[99]
Anemia in celiac disease	Oligofructose-enriched inulin	Orally; supplement	Decreased serum hepcidin conc. - ↑ iron absorption	[100]
Vitro	*Lactobacillus plantarum* CIDCA * 83114 and pectin	Capsules	↑ iron absorption	[101]
Iron-depleted	Galacto-oligosaccharides	Orally	↑ iron absorption	[102]
Anemia (IDA)	Galacto-oligosaccharides	Injection	↑ iron absorption	[103]
Anemia (IDA)	Inulin and oligofructose	Orally; dietary fibre	↑ the expression of the divalent metal transporter protein in the caecum and oligofructose decreased the expression of the protein ferroportin in the duodenum Helps regulate of intestinal iron absorption	[8]
Anemia (IDA)	Galacto-oligosaccharides	Orally; maize porridge fortified with a micronutrient powder (ferrous fumarate + sodium iron + galacto-oligosaccharide)	↑ fractional iron absorption (62%) Improved the relative iron bioavailability	[104]
Healthy (Kenyan mothers)	Galacto-oligosaccharides	Orally; micronutrient powder (ferrous fumarate + sodium iron EDTA ** + galacto-oligosaccharides)	Modulate the infant gut microbiota response to fortificant iron	[105]
Anemia (IDA)	*B. bifidum*, *B. longum* galactooligosaccharides	Orally; powder follow-up infant formula	↑ the apparent iron absorption or retention	[60]
Low iron status	Inulin	Orally; cooked rice and a pureed, boiled vegetable sauce	↑ iron absorption	[106]
Anemia (IDA)	inulin, polidextrose, arabic gum, and guar gum	Orally in yoghurt, 2 g per day	↑ heme iron bioavailability not influence non-heme iron bioavailability	[107]

* CIDCA—Centro de Investigación y Desarrollo en Criotecnología de Alimentos. ** EDTA—Ethylenediaminetetraacetic acid.

## References

[1] Boccio J.R., Iyengar V. (2003). Iron deficiency: Causes, consequences, and strategies to overcome this nutritional problem. Biol. Trace Elem. Res..

[2] Goddard A.F., James M.W., McIntyre A.S., Scott B.B. (2011). Guidelines for the management of iron deficiency anaemia. Gut.

[3] Miller J.L. (2013). Iron deficiency anemia: A common and curable disease. Cold Spring Harb. Perspect Med..

[4] Chiplonkar S.A., Agte V.V. (2006). Statistical model for predicting non-heme iron bioavailability from vegetarian meals. Int. J. Food Sci. Nutr..

[5] Reddy M.B. (2005). Algorithms to assess non-heme iron bioavailability. Int. J. Vitam. Nutr. Res..

[6] Vonderheid S.C., Tussing-Humphreys L., Park C., Pauls H., Hemphill N.O., LaBomascus B., McLeod A., Koenig M.D. (2019). A Systematic Review and Meta-Analysis on the Effects of Probiotic Species on Iron Absorption and Iron Status. Nutrients.

[7] Hoppe M., Önning G., Berggren A., Hulthén L. (2015). Probiotic strain Lactobacillus plantarum 299v increases iron absorption from an iron-supplemented fruit drink: A double-isotope cross-over single-blind study in women of reproductive age. Brit. J. Nutr..

[8] Marciano R., Santamarina A.B., de Santana A.A., Silva M.D.L.C., Amancio O.M.S., do Nascimento C.M.D.P.O., Oyama L.M., de Morais M.B. (2015). Effects of prebiotic supplementation on the expression of proteins regulating iron absorption in anaemic growing rats. Brit. J. Nutr..

[9] Gupta A. (2017). Iron Metabolism in Human Body. Nutritional Anemia in Preschool Children.

[10] Camaschella C. (2015). Iron-deficiency anemia. N. Engl. J. Med..

[11] Wilson A.S., Koller K.R., Ramaboli M.C., Nesengani L.T., Ocvirk S., Chen C.X., Flanagan C.A., Sapp F.R., Merritt Z.T., Bhatti F. (2020). Diet and the Human Gut Microbiome: An International Review. Dig. Dis. Sci..

[12] Andrews S.C., Robinson A.K., Rodríguez-Quiñones F. (2003). Bacterial iron homeostasis. FEMS Microbiol. Rev..

[13] Braun V., Hantke K. (2011). Recent insights into iron import by bacteria. Curr. Opin. Chem. Biol..

[14] Xi R., Wang R., Wang Y., Xiang Z., Su Z., Cao Z., Xu X., Zheng X., Li J. (2019). Comparative analysis of the oral microbiota between iron-deficiency anaemia (IDA) patients and healthy individuals by high-throughput sequencing. BMC Oral Health.

[15] Beasley F.C., Marolda C.L., Cheung J., Buac S., Heinrichs D.E. (2011). Staphylococcus aureus transporters Hts, Sir, and Sst capture iron liberated from human transferrin by Staphyloferrin A, Staphyloferrin B, and catecholamine stress hormones, respectively, and contribute to virulence. Infect. Immun..

[16] Cherayil B.J., Ellenbogen S., Shanmugam N.N. (2011). Iron and intestinal immunity. Curr. Opin. Gastroenterol..

[17] van de Lagemaat M., Amesz E.M., Schaafsma A., Lafeber H.N. (2014). Iron deficiency and anemia in iron-fortified formula and human milk-fed preterm infants until 6 months post-term. Eur. J. Nutr..

[18] Shubham K., Anukiruthika T., Dutta S., Kashyap A.V., Moses J.A., Anandharamakrishnan C. (2020). Iron deficiency anemia: A comprehensive review on iron absorption, bioavailability and emerging food fortification approaches. Trends Food Sci. Technol..

[19] Bierings M., Clayton P.T., Houwen R.H. (2012). Disorders in the transport of copper, iron, magnesium, manganese, selenium and zinc. Inborn Metabolic Diseases.

[20] Jansen V. (2019). Diagnosis of anemia—A synoptic overview and practical approach. Transfus. Apher. Sci..

[21] Kowdley K.V., Brown K.E., Ahn J., Sundaram V. (2019). ACG Clinical Guideline: Hereditary Hemochromatosis. Am. J. Gastroenterol..

[22] Arredondo M., Núñez M.T. (2005). Iron and copper metabolism. Mol. Asp. Med..

[23] Fan Y., Dhaliwal H.K., Menon A.V., Chang J., Choi J.E., Amiji M.M., Kim J. (2019). Site-specific intestinal DMT1 silencing to mitigate iron absorption using pH-sensitive multi-compartmental nanoparticulate oral delivery system. Nanomed. Nanotechnol. Biol. Med..

[24] Hentze M.W., Muckenthaler M.U., Galy B., Camaschella C. (2010). Two to Tango: Regulation of Mammalian Iron Metabolism. Cell.

[25] Pandey S.S., Patnana P.K., Lomada S.K., Tomar A., Chatterjee S. (2016). Co-regulation of Iron Metabolism and Virulence Associated Functions by Iron and XibR, a Novel Iron Binding Transcription Factor, in the Plant Pathogen Xanthomonas. PLoS Pathog..

[26] Shen J., Sheng X., Chang Z., Wu Q., Wang S., Xuan Z., Li D., Wu Y., Shang Y., Kong X. (2014). Iron Metabolism Regulates p53 Signaling through Direct Heme-p53 Interaction and Modulation of p53 Localization, Stability, and Function. Cell Rep..

[27] Wang Y., Yu L., Ding J., Chen Y. (2019). Iron Metabolism in Cancer. Int. J. Mol. Sci..

[28] Yaskolka Meir A., Tsaban G., Zelicha H., Rinott E., Kaplan A., Youngster I., Rudich A., Shelef I., Tirosh A., Brikner D. (2019). A green-Mediterranean diet, supplemented with Mankai duckweed, preserves iron-homeostasis in humans and is efficient in reversal of anemia in rats. J. Nutr..

[29] Das N.K., Schwartz A.J., Barthel G., Inohara N., Liu Q., Sankar A., Hill D.R., Ma X., Lamberg O., Schnizlein M.K. (2020). Microbial Metabolite Signaling Is Required for Systemic Iron Homeostasis. Cell Metab..

[30] Jia H.X., Han J.H., Li H.Z., Liang D., Deng T.T., Chang S.Y. (2016). Mineral Intake in Urban Pregnant Women from Base Diet, Fortified Foods, and Food Supplements: Focus on Calcium, Iron, and Zinc. Biomed. Environ. Sci..

[31] World Health Organization (2015). Haemoglobin Concentrations for the Diagnosis of Anaemia and Assessment of Severity.

[32] Theml H., Diem H., Haferlach T. (2004). Color Atlas of Hematology.

[33] Helmyati S., Rahayu E.S., Kandarina B.J.I., Juffrie M. (2020). No Difference between Iron Supplementation Only and Iron Supplementation with Synbiotic Fermented Milk on Iron Status, Growth, and Gut Microbiota Profile in Elementary School Children with Iron Deficiency. Curr. Nutr. Food Sci..

[34] Jaeggi T., Kortman G.A.M., Moretti D., Chassard C., Holding P., Dostal A., Boekhorst J., Timmerman H.M., Swinkels D.W., Tjalsma H. (2015). Iron fortification adversely affects the gut microbiome, increases pathogen abundance and induces intestinal inflammation in Kenyan infants. Gut.

[35] Da Silva W.R., Silveira L., Fernandes A.B. (2019). Diagnosing sickle cell disease and iron deficiency anemia in human blood by Raman spectroscopy. Lasers Med. Sci..

[36] Saini R.K., Nile S.H., Keum Y.-S. (2016). Food science and technology for management of iron deficiency in humans: A review. Trends Food Sci. Technol..

[37] Cammarota G., Ianiro G., Bibbò S., Gasbarrini A. (2014). Gut microbiota modulation: Probiotics, antibiotics or fecal microbiota transplantation?. Intern. Emerg. Med..

[38] Gabbianelli R., Damiani E. (2018). Epigenetics and neurodegeneration: Role of early-life nutrition. J. Nutr. Biochem..

[39] Kim Y.S., Unno T., Kim B.-Y., Park M.-S. (2020). Sex Differences in Gut Microbiota. World J. Mens Health.

[40] Ursell L.K., Metcalf J.L., Parfrey L.W., Knight R. (2012). Defining the human microbiome. Nutr. Rev..

[41] Tungland B., Tungland B. (2018). Chapter 9—Dysbiosis of the Microbiota: Therapeutic Strategies Utilizing Dietary Modification, Pro- and Prebiotics and Fecal Transplant Therapies in Promoting Normal Balance and Local GI Functions. Human Microbiota in Health and Disease.

[42] Kim S.R., Kim K., Lee S.A., Kwon S.O., Lee J.-K., Keum N., Park S.M. (2019). Effect of Red, Processed, and White Meat Consumption on the Risk of Gastric Cancer: An Overall and Dose–Response Meta-Analysis. Nutrients.

[43] Boran P., Baris H.E., Kepenekli E., Erzik C., Soysal A., Dinh D.M. (2020). The impact of vitamin B12 deficiency on infant gut microbiota. Eur. J. Pediatr..

[44] Deschemin J.C., Noordine M.L., Remot A., Willemetz A., Afif C., Canonne-Hergaux F., Langella P., Karim Z., Vaulont S., Thomas M. (2016). The microbiota shifts the iron sensing of intestinal cells. FASEB J..

[45] Forth W., Rummel W. (1973). Iron absorption. Physiol. Rev..

[46] Wollenberg P., Rummel W. (1987). Dependence of intestinal iron absorption on the valency state of iron. N-S Arch. Pharmacol..

[47] Dietzfelbinger H. (1987). Bioavailability of bi-and trivalent oral iron preparations. Investigations of iron absorption by postabsorption serum iron concentrations curves. Arzneimittel-Forschung.

[48] Bezkorovainy A. (1989). Biochemistry of nonheme iron in man. I. Iron proteins and cellular iron metabolism. Clin. Physiol. Biochem..

[49] Cremonesi P., Acebron A., Raja K.B., Simpson R.J. (2002). Iron absorption: Biochemical and molecular insights into the importance of iron species for intestinal uptake. Pharmacol. Toxicol..

[50] Simonyté Sjödin K., Domellöf M., Lagerqvist C., Hernell O., Lönnerdal B., Szymlek-Gay E.A., Sjödin A., West C.E., Lind T. (2019). Administration of ferrous sulfate drops has significant effects on the gut microbiota of iron-sufficient infants: A randomised controlled study. Gut.

[51] Balamurugan R., Mary R.R., Chittaranjan S., Jancy H., Shobana Devi R., Ramakrishna B.S. (2010). Low levels of faecal lactobacilli in women with iron-deficiency anaemia in south India. Brit. J. Nutr..

[52] Jahani-Sherafat S., Alebouyeh M., Moghim S., Ahmadi Amoli H., Ghasemian-Safaei H. (2018). Role of gut microbiota in the pathogenesis of colorectal cancer; a review article. Gastroenterol. Hepatol. Bed. Bench..

[53] Louis P., Hold G.L., Flint H.J. (2014). The gut microbiota, bacterial metabolites and colorectal cancer. Nat. Rev. Microbiol..

[54] Gagniere J., Raisch J., Veziant J., Barnich N., Bonnet R., Buc E., Bringer M.A., Pezet D., Bonnet M. (2016). Gut microbiota imbalance and colorectal cancer. World J. Gastroenterol..

[55] Gao Z., Guo B., Gao R., Zhu Q., Qin H. (2015). Microbiota disbiosis is associated with colorectal cancer. Front. Microbiol..

[56] Ng O. (2016). Iron, microbiota and colorectal cancer. Wien. Med. Wochenschr..

[57] Dostal A., Baumgartner J., Riesen N., Chassard C., Smuts C.M., Zimmermann M.B., Lacroix C. (2014). Effects of iron supplementation on dominant bacterial groups in the gut, faecal SCFA and gut inflammation: A randomised, placebo-controlled intervention trial in South African children. Brit. J. Nutr..

[58] Dostal A., Chassard C., Hilty F.M., Zimmermann M.B., Jaeggi T., Rossi S., Lacroix C. (2012). Iron depletion and repletion with ferrous sulfate or electrolytic iron modifies the composition and metabolic activity of the gut microbiota in rats. J. Nutr..

[59] Buhnik-Rosenblau K., Moshe-Belizowski S., Danin-Poleg Y., Meyron-Holtz E.G. (2012). Genetic modification of iron metabolism in mice affects the gut microbiota. BioMetals.

[60] Perez-Conesa D., Lopez G., Ros G. (2007). Effect of probiotic, prebiotic and synbiotic follow-up infant formulas on iron bioavailability in rats. Food Sci. Technol. Int..

[61] Collins J.F., Flores S.R., Wang X., Anderson G.J. (2018). Mechanisms and Regulation of Intestinal Iron Transport. Physiology of the Gastrointestinal Tract.

[62] González A., Gálvez N., Martín J., Reyes F., Pérez-Victoria I., Dominguez-Vera J.M. (2017). Identification of the key excreted molecule by Lactobacillus fermentum related to host iron absorption. Food Chem..

[63] Gan E.K., Powell L.W., Olynyk J.K. (2011). Natural History and Management of HFE-Hemochromatosis. Semin. Liver Dis..

[64] Oh C.-K., Moon Y. (2019). Dietary and Sentinel Factors Leading to Hemochromatosis. Nutrients.

[65] Camaschella C., Poggiali E. (2011). Inherited disorders of iron metabolism. Curr. Opin. Pediatrics.

[66] Pantopoulos K. (2018). Inherited Disorders of Iron Overload. Front. Nutr..

[67] Gulec S., Anderson G.J., Collins J.F. (2014). Mechanistic and regulatory aspects of intestinal iron absorption. Am. J. Physiol. Gastrointest. Liver Physiol..

[68] Aslam M.F., Frazer D.M., Faria N., Bruggraber S.F.A., Wilkins S.J., Mirciov C., Powell J.J., Anderson G.J., Pereira D.I.A. (2014). Ferroportin mediates the intestinal absorption of iron from a nanoparticulate ferritin core mimetic in mice. FASEB J..

[69] Carvalho L., Brait D., Vaz M., Lollo P., Morato P., Oesterreich S., Raposo J., Freitas K. (2017). Partially Hydrolyzed Guar Gum Increases Ferroportin Expression in the Colon of Anemic Growing Rats. Nutrients.

[70] Liu B.D., Pan X.H., Liu Z.H., Han M.L., Xu G.H., Dai X.S., Wang W., Zhang H.B., Xie L.W. (2020). Fecal microbiota as a noninvasive biomarker to predict the tissue iron accumulation in intestine epithelial cells and liver. FASEB J..

[71] Kalipatnapu S., Kuppuswamy S., Venugopal G., Kaliaperumal V., Ramadass B. (2017). Fecal total iron concentration is inversely associated with fecal Lactobacillus in preschool children. J. Gastroenterol. Hepatol..

[72] Buret A.G., Motta J.-P., Allain T., Ferraz J., Wallace J.L. (2019). Pathobiont release from dysbiotic gut microbiota biofilms in intestinal inflammatory diseases: A role for iron?. J. Biomed. Sci..

[73] Martınez-Navarrete N., Camacho M.M., Martınez-Lahuerta J., Martınez-Monzó J., Fito P. (2002). Iron deficiency and iron fortified foods—A review. Food Res. Int..

[74] Brazaca S.G.C., da Silva F.C. (2003). Enhancers and inhibitors of iron availability in legumes. Plant Foods Hum. Nutr..

[75] Record I.R., McInerney J.K., Dreosti I.E. (1996). Black tea, green tea, and tea polyphenols. Biol. Trace Elem. Res..

[76] Milne D.B., Canfield W.K., Mahalko J.R., Sandstead H.H. (1984). Effect of oral folic acid supplements on zinc, copper, and iron absorption and excretion. Am. J. Clin. Nutr..

[77] Shu E., Ogbodo S. (2005). Role of Ascorbic Acid in the Prevention of Iron-Deficiency Anaemia in Pregnancy. Biomed. Res..

[78] Martínez-Torres C., Romano E., Layrisse M. (1981). Effect of cysteine on iron absorption in man. Am. J. Clin. Nutr..

[79] García-Casal M.N., Layrisse M., Solano L., Barón M.A., Arguello F., Llovera D., Ramírez J., Leets I., Tropper E. (1998). Vitamin A and β-Carotene Can Improve Nonheme Iron Absorption from Rice, Wheat and Corn by Humans. J. Nutr..

[80] Amos A., Alvan A., Florence A. (2020). The Anti-nutritional Effect of Phytate on Zinc, Iron and Calcium Bioavailabilities of Some Cereals Staple Foods in Zaria, Nigeria. Eur. J. Nutr. Food Saf..

[81] Rosen G.M., Morrissette S., Larson A., Stading P., Griffin K.H., Barnes T.L. (2019). Use of a Probiotic to Enhance Iron Absorption in a Randomized Trial of Pediatric Patients Presenting with Iron Deficiency. J. Pediatr..

[82] Skrypnik K., Bogdanski P., Schmidt M., Suliburska J. (2019). The Effect of Multispecies Probiotic Supplementation on Iron Status in Rats. Biol. Trace Elem. Res..

[83] Helmyati S., Sudargo T., Kandarina I., Yuliati E., Wisnusanti S.U., Puspitaningrum V.A.D., Juffrie M. (2016). Tempeh extract fortified with iron and synbiotic as a strategy against anemia. Int. Food Res. J..

[84] Deriu E., Liu J.Z., Pezeshki M., Edwards R.A., Ochoa R.J., Contreras H., Libby S.J., Fang F.C., Raffatellu M. (2013). Probiotic bacteria reduce salmonella typhimurium intestinal colonization by competing for iron. Cell Host Microbe.

[85] Adeyanju A.A., Kruger J., Taylor J.R., Duodu K.G. (2019). Effects of different souring methods on the protein quality and iron and zinc bioaccessibilities of non-alcoholic beverages from sorghum and amaranth. Int. J. Food Sci. Technol..

[86] Adiki S.K., Perla C.K., Saha G., Katakam P., Theendra V. (2019). Enhancement in Iron Absorption on Intake of Chemometrically Optimized Ratio of Probiotic Strain Lactobacillus plantarum 299v with Iron Supplement Pearl Millet. Biol. Trace Elem. Res..

[87] El-Azeem A.S.A., Hegazy A.M., Badawy I., Ibrahim G.A., El-Shafei K., El-Sayed H.S., Sharaf O.M. (2016). Effectiveness of Functional Wheat-Fermented Milk Beverage against Tannic Acid Induced Anemia. Res. J. Pharm. Biol. Chem. Sci..

[88] Garcés V., Rodríguez-Nogales A., González A., Gálvez N., Rodríguez-Cabezas M.E., García-Martin M.L., Gutiérrez L., Rondón D., Olivares M., Gálvez J. (2018). Bacteria-carried iron oxide nanoparticles for treatment of anemia. Bioconj. Chem..

[89] Hoppe M., Önning G., Hulthén L. (2017). Freeze-dried Lactobacillus plantarum 299v increases iron absorption in young females—Double isotope sequential single-blind studies in menstruating women. PLoS ONE.

[90] Khodaii Z., Zadeh M.N., Kamali J., Natanzi M.M. (2019). Enhanced iron absorption from lactic acid fermented bread (an in vivo/ex vivo study). Gene Rep..

[91] Korčok D.J., Tršić-Milanoviće N., Ivanović N., Đorđević B. (2018). Development of Probiotic Formulation for the Treatment of Iron Deficiency Anemia. Chem. Pharm. Bull..

[92] Scheers N., Rossander-Hulthen L., Torsdottir I., Sandberg A.-S. (2016). Increased iron bioavailability from lactic-fermented vegetables is likely an effect of promoting the formation of ferric iron (Fe 3+). Eur. J. Nutr..

[93] Skrypnik K., Bogdański P., Sobieska M., Suliburska J. (2019). The effect of multistrain probiotic supplementation in two doses on iron metabolism in obese postmenopausal women: A randomized trial. Food Funct..

[94] Tungland B., Tungland B. (2018). Chapter 5—Direct Physiological Effects on Local Gi and Indirect Systemic Effects of Prebiotic Fructan Treatment, and its Role in Disease Prevention and Therapy. Human Microbiota in Health and Disease.

[95] Rizwan Ahmad A.M., Ahmed W., Iqbal S., Mushtaq M.H., Anis R.A. (2020). Iron and prebiotic fortified flour improves the immune function of iron deficient women of childbearing age. Pak. J. Pharm. Sci..

[96] Castro L.C.V., Costa N.M.B., Sant’Anna H.M.P., Ferreira C.L.d.L.F., Franceschini S.d.C.d.C. (2017). Improvement the nutritional status of pre-school children following intervention with a supplement containing iron, zinc, copper, vitamin A, vitamin C and prebiotic. Ciên. Saú. Colet..

[97] Christides T., Ganis J.C., Sharp P.A. (2018). In vitro assessment of iron availability from commercial Young Child Formulae supplemented with prebiotics. Eur. J. Nutr..

[98] Dabour N., Dyab N., Kheadr E. (2019). Iron fortification of reduced-fat bioyoghurt containing either short-or long-chain inulin. Int. J. Dairy Technol..

[99] Sazawal S., Dhingra U., Hiremath G., Sarkar A., Dhingra P., Dutta A., Menon V.P., Black R.E. (2010). Effects of Bifidobacterium lactis HN019 and prebiotic oligosaccharide added to milk on iron status, anemia, and growth among children 1 to 4 years old. J. Ped. Gastroenterol. Nutr..

[100] Feruś K., Drabińska N., Krupa-Kozak U., Jarocka-Cyrta E. (2018). A randomized, placebo-controlled, pilot clinical trial to evaluate the effect of supplementation with prebiotic Synergy 1 on iron homeostasis in children and adolescents with celiac disease treated with a gluten-free diet. Nutrients.

[101] Ghibaudo F., Gerbino E., Copello G.J., Campo Dall’ Orto V., Gómez-Zavaglia A. (2019). Pectin-decorated magnetite nanoparticles as both iron delivery systems and protective matrices for probiotic bacteria. Colloids Surf. B Biointerfaces.

[102] Jeroense F.M., Michel L., Zeder C., Herter-Aeberli I., Zimmermann M.B. (2019). Consumption of galacto-oligosaccharides increases iron absorption from ferrous fumarate: A stable iron isotope study in iron-depleted young women. J. Nutr..

[103] Laparra J.M., Díez-Municio M., Herrero M., Moreno F.J. (2014). Structural differences of prebiotic oligosaccharides influence their capability to enhance iron absorption in deficient rats. Food Funct..

[104] Paganini D., Uyoga M.A., Cercamondi C.I., Moretti D., Mwasi E., Schwab C., Bechtler S., Mutuku F.M., Galetti V., Lacroix C. (2017). Consumption of galacto-oligosaccharides increases iron absorption from a micronutrient powder containing ferrous fumarate and sodium iron EDTA: A stable-isotope study in Kenyan infants. Am. J. Clin. Nutr..

[105] Paganini D., Uyoga M.A., Kortman G.A., Boekhorst J., Schneeberger S., Karanja S., Hennet T., Zimmermann M.B. (2019). Maternal Human Milk Oligosaccharide Profile Modulates the Impact of an Intervention with Iron and Galacto-Oligosaccharides in Kenyan Infants. Nutrients.

[106] Petry N., Egli I., Chassard C., Lacroix C., Hurrell R. (2012). Inulin modifies the bifidobacteria population, fecal lactate concentration, and fecal pH but does not influence iron absorption in women with low iron status. Am. J. Clin. Nutr..

[107] Weinborn V., Valenzuela C., Olivares M., Arredondo M., Weill R., Pizarro F. (2017). Prebiotics increase heme iron bioavailability and do not affect non-heme iron bioavailability in humans. Food Funct..

[108] Maawia K., Iqbal S., Qamar T.R., Rafiq P., ullah A., Ahmad M.-U.-D. (2016). Production of impure prebiotic galacto-oligosaccharides and their effect on calcium, magnesium, iron and zinc absorption in Sprague-Dawley rats. Pharma Nutr..

[109] Pitarresi G., Tripodo G., Cavallaro G., Palumbo F.S., Giammona G. (2008). Inulin–iron complexes: A potential treatment of iron deficiency anaemia. Eur. J. Pharm. Biopharm..

[110] Laparra J.M., Tako E., Glahn R.P., Miller D.D. (2008). Supplemental inulin does not enhance iron bioavailability to Caco-2 cells from milk- or soy-based, probiotic-containing, yogurts but incubation at 37 °C does. Food Chem..

[111] Asemi Z., Aarabi M.H., Hajijafari M., Alizadeh S.A., Razzaghi R., Mazoochi M., Esmaillzadeh A. (2017). Effects of synbiotic food consumption on serum minerals, liver enzymes, and blood pressure in patients with type 2 diabetes: A double-blind randomized cross-over controlled clinical trial. Int. J. Prev. Med..

